# Facile Tailoring of Metal‐Organic Frameworks for Förster Resonance Energy Transfer‐Driven Enhancement in Perovskite Photovoltaics

**DOI:** 10.1002/advs.202307476

**Published:** 2024-03-06

**Authors:** Xiao Liang, Hai‐lun Xia, Jin Xiang, Fei Wang, Jing Ma, Xianfang Zhou, Hao Wang, Xiao‐Yuan Liu, Quanyao Zhu, Haoran Lin, Jun Pan, Mingjian Yuan, Gang Li, Hanlin Hu

**Affiliations:** ^1^ Hoffmann Institute of Advanced Materials Shenzhen Polytechnic 7098 Liuxian Boulevard Shenzhen 518055 China; ^2^ State Key Laboratory of Advanced Technology for Materials Synthesis and Processing School of Materials Science and Engineering Wuhan University of Technology Wuhan 430070 China; ^3^ Medical Intelligence and Innovation Academy Southern University of Science and Technology Hospital Shenzhen 518055 China; ^4^ College of Materials Science and Engineering Zhejiang University of Technology Hangzhou 310014 China; ^5^ Renewable Energy Conversion and Storage Center (RECAST) College of Chemistry Nankai University Tianjin 300071 China; ^6^ Department of Electronic and Information Engineering Research Institute for Smart Energy (RISE) The Hong Kong Polytechnic University Hung Hom Kowloon Hong Kong 999077 China

**Keywords:** Förster resonance energy transfer, metal‐organic frameworks, perovskite photovoltaics

## Abstract

Förster resonance energy transfer (FRET) has demonstrated its potential to enhance the light energy utilization ratio of perovskite solar cells by interacting with metal‐organic frameworks (MOFs) and perovskite layers. However, comprehensive investigations into how MOF design and synthesis impact FRET in perovskite systems are scarce. In this work, nanoscale HIAM‐type Zr‐MOF (HIAM‐4023, HIAM‐4024, and HIAM‐4025) is meticulously tailored to evaluate FRET's existence and its influence on the perovskite photoactive layer. Through precise adjustments of amino groups and acceptor units in the organic linker, HIAM‐MOFs are synthesized with the same topology, but distinct photoluminescence (PL) emission properties. Significant FRET is observed between HIAM‐4023/HIAM‐4024 and the perovskite, confirmed by spectral overlap, fluorescence lifetime decay, and calculated distances between HIAM‐4023/HIAM‐4024 and the perovskite. Conversely, the spectral overlap between the PL emission of HIAM‐4025 and the perovskite's absorption spectrum is relatively minimal, impeding the energy transfer from HIAM‐4025 to the perovskite. Therefore, the HIAM‐4023/HIAM‐4024‐assisted perovskite devices exhibit enhanced EQE via FRET processes, whereas the HIAM‐4025 demonstrates comparable EQE to the pristine. Ultimately, the HIAM‐4023‐assisted perovskite device achieves an enhanced power conversion efficiency (PCE) of 24.22% compared with pristine devices (PCE of 22.06%) and remarkable long‐term stability under ambient conditions and continuous light illumination.

## Graphical abstract

Nanoscale HIAM‐type Zr‐metal‐organic framework (MOF) is meticulously tailored to evaluate Förster resonance energy transfer (FRET's) existence. Significant FRET to the perovskite is confirmed via spectral overlap and fluorescence decay, with calculated proximities of HIAM‐4023/4024 conducive to energy transfer. HIAM‐4025, however, shows minimal spectral overlap with perovskite's absorption, limiting energy transfer. The final device improves optoelectronic properties by over 24%.

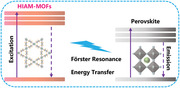

## 1 Introduction

Typically, energy transfer between a donor and an acceptor involves three distinct modes: 1) Förster resonance energy transfer (FRET), 2) nanosurface energy transfer (NSET), and 3) nanovolume energy transfer (NVET).^[^ ^1^, ^2^ ^]^ FRET is a nonradiative process wherein energy is transferred from a donor material to a proximal appropriate acceptor material through dipole–dipole interactions. The efficiency of FRET depends on the distance between the donor and acceptor (1–10 nm) and is contingent upon the spectral overlap between the donor's emission spectrum and the acceptor's absorption spectrum.^[^ ^3^, ^4^, ^5^, ^6^, ^7^, ^8^, ^9^, ^10^, ^11^, ^12^ ^]^ However, NSET/NVET does not necessitate spectral overlap between donor emission and acceptor absorption, enabling these processes to measure distances greater than the typical Förster distance.^[^ ^13^ ^]^ The pioneering work of Yun et al.^[^ ^14^ ^]^ reported the first successful development of a NSET process utilizing dipole‐surface type energy transfer from molecular dipoles to nano‐metal surfaces, even at distances up to 22 nm. Lupton et al.^[^ ^15^ ^]^ discovered energy transfer from nanocrystals to single dye molecules that do not rely on the spectral overlap between donor and acceptor. Instead, it involves a non‐resonant excitation energy transfer, implying strong electron–phonon coupling of the carriers constituting the exciton, as opposed to the dipole‐dipole coupling in FRET processes. Chen et al.^[^ ^16^ ^]^ highlighted the absorption spectrum of perovskites overlaps with the PL spectrum of Eu‐TCPP MOF, suggesting the possibility of FRET between Eu‐TCPP MOF and perovskite layers. Similarly, Tan et al.^[^ ^17^ ^]^ underscored that MOFs serve as excellent platforms for triggering FRET mechanisms. Consequently, we have summarized the recently reported energy transfer mechanisms between MOF and perovskite, as shown in Table S1 (Supporting Information). Upon careful examination, we observe that FRET typically occurs between MOF and perovskite. Building on this insight, we delve into the discussion of FRET occurring between MOF and perovskite, aligning with the established literature on this subject.

Currently, FRET is used in perovskite photovoltaics, exerting a significant impact on their photovoltaic performance. It permits the nanoscale monitoring of molecular interactions, supporting an in‐depth comprehension of the complex processes underlying perovskite photovoltaics, as well as the optimization of device design and engineering strategies for the manufacture of efficient and durable perovskite solar cells (PSCs).^[^ ^18^, ^19^, ^20^, ^21^ ^]^ FRET is particularly effective in energy transfer among fluorescent species, optimizing light energy absorption and utilization within perovskite materials. Through fine‐tuning FRET processes, the spatial and spectral overlap between energy donor and acceptor sections can be tailored, thereby maximizing the energy transfer efficiency.^[^ ^22^, ^23^ ^]^ This approach facilitates the rational and precise design of functional materials by integrating diverse molecular components into perovskite structures.^[^ ^24^, ^25^ ^]^ Importantly, researchers recognized the distinctive benefits of MOFs as mediators and facilitators of FRET processes in perovskite systems, which offer opportunities for boosting light harvesting and charge carrier dynamics in perovskite photovoltaics.^[^ ^26^, ^27^ ^]^ Lee and co‐workers^[^ ^28^ ^]^ reported that FRET between MOF and perovskite might occur if it has a sufficient PL intensity and quantum yield, which is helpful for enhancing the resultant photocurrent of derived devices. Zhou and co‐workers^[^ ^29^ ^]^ discovered that the In‐BTC‐assisted PSC indicates a significantly improved photo‐response, which was attributed to the FRET between In‐BTC and perovskite owing to the high overlap between the PL emission spectrum of In‐BTC and the UV–Vis absorption spectrum of perovskite. Dou et al.^[^ ^30^ ^]^ reported that the excitation peak of the Eu‐MOF overlaps accurately with the perovskite absorption, satisfying the criteria for efficient FRET from the Eu‐MOF to the adjacent perovskite layer. This result suggested that high‐energy particles could be filtered by the Eu‐MOF layer, which emits visible‐spectrum photons that are then utilized by the perovskite absorber below to increase the light energy utilization ratio of the corresponding PSCs via down‐conversion. However, comprehensive studies addressing the influence of MOF design and synthesis on FRET in perovskite systems remain scarce.

In this article, we have synthesized nanoscale (approximately 50–70 nm) HIAM‐type Zr‐MOF (HIAM‐4023, HIAM‐4024, and HIAM‐4025) materials (HIAM = Hoffmann Institute of Advanced Materials; 40 = Zirconium) with tunable emission wavelengths via tailoring and synthesizing three distinct organic linkers with distinct emission properties and subsequently combining them with Zr_6_ clusters. Supplemental Information supplies detailed synthetic routes for the HIAM‐MOF.^[^ ^31^, ^32^ ^]^ Interestingly, we observed relevant FRET between HIAM‐4023/HIAM‐4024, and the perovskite, as evidenced by pronounced spectral overlap, fluorescence lifetime decay, and calculated distances between HIAM‐4023/HIAM‐4024, and the perovskite, meeting the prerequisites for FRET occurrence. In comparison, the PL emission spectrum of HIAM‐4025 closely overlaps that of the perovskite, whereas the spectral overlap between its PL emission and the perovskite's absorption spectrum is relatively limited, impeding efficient energy transfer from HIAM‐4025 to the perovskite. Thus, the HIAM‐4023 and HIAM‐4024 assisted perovskite cells exhibit enhanced EQE via FRET, while the HIAM‐4025 shows similar EQE to the pristine devices. Furthermore, the HIAM‐MOF‐assisted perovskite thin films demonstrate superior film quality and a complete conversion of PbI_2_ into perovskite material. Eventually, the HIAM‐4023‐assisted photovoltaic device shows a high PCE of 24.22%, with 9.8% enhancement compared with the pristine device, and the unencapsulated PSCs exhibit enhanced long‐term stability under ambient and continuous light‐soaking conditions. This study provides valuable insights into the occurrence of FRET in perovskite facilitated by MOFs.

## 2 Results and Discussion

**Figure 1:**
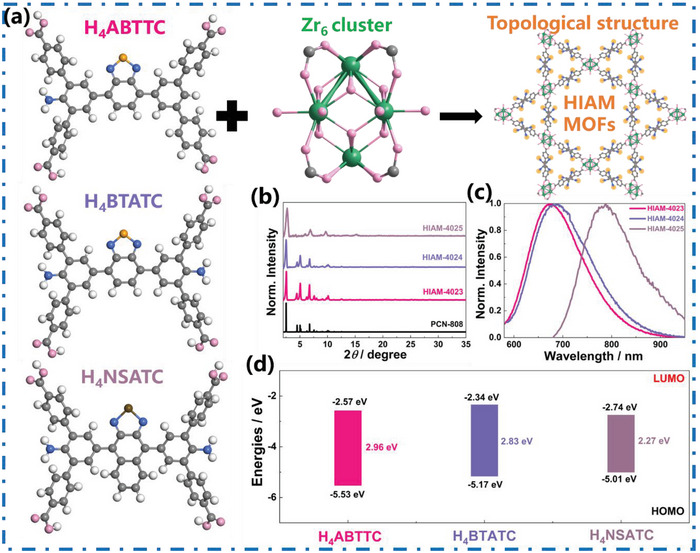
a) The topological structure of HIAM‐MOFs was synthesized via the combination of Zr_6_ clusters with the organic linkers of H_4_ABTTC, H_4_BTATC, and H_4_NSATC. b) The powder X‐ray diffraction (PXRD) patterns of PCN‐808 and prepared HIAM‐4023, HIAM‐4024, and HIAM‐4025. c) The normalized solid‐state photoluminescence (PL) emission spectra of HIAM‐4023, HIAM‐4024, and HIAM‐4025. d) The HOMO–LUMO energy levels of H_4_ABTTC, H_4_BTATC, and H_4_NSATC were computationally determined.

In our investigation, we employed a meticulous approach to study FRET between HIAM‐MOFs with tailored emission wavelengths and perovskite material. To achieve this, we designed distinct linker molecules by adjusting the number of amino groups and modifying acceptor units in the organic linkers, thus impacting the emission/absorption properties of the resulting MOFs.^[^ ^31^ ^]^ This led to the synthesis of three HIAM‐MOFs: HIAM‐4023, HIAM‐4024, and HIAM‐4025, with similar topological structures. The molecular structures of three organic linkers, 2′‐amino‐5′‐(7‐(4,4′′‐dicarboxy ‐[1,1′:3′,1′′‐terphenyl]−5′‐yl)benzo[c][1,2,5] thiadiazol‐4‐yl)‐[1,1′:3′,1′′‐terphenyl]−4,4′′‐dicarboxylic acid (H_4_ABTTC) with one amino group, 5′,5′′′′‐(benzo[c][1,2,5] thiadiazole‐4,7‐diyl)bis(2′‐amino‐[1,1′:3′,1′′‐terphenyl] −4,4′′‐dicarboxylic acid) (H_4_BTATC) with two amino groups, and 5′,5′′′′‐(naphtho [2,3‐c][1,2,5] selenadiazole‐4,9‐diyl) bis (2′‐amino‐[1,1′:3′,1′′‐terphenyl] −4,4′′‐ dicarboxylic acid) (H_4_NSATC) with the changed acceptor, were designed and provided (**Figure** **1**, left and Figure S1, Supporting Information). The organic linkers H_4_ABTTC, H_4_BTATC, and H_4_NSATC were employed to react with Zr_6_ clusters to form the corresponding MOFs, HIAM‐4023, HIAM‐4024, and HIAM‐4025, respectively (Figure 1a). The remaining consistency between the experimental powder X‐ray diffraction (PXRD) patterns of HIAM‐MOF and PCN‐808 supports the high phase purity of the synthesized material and the isoreticular nature (Figure 1b). Moreover, the powder of HIAM‐4024 exhibits photoluminescence (PL) emissions with a peak maximum at 688 nm, indicating a 12 nm bathochromic shift relative to HIAM‐4023. This red shift in the emission energy/wavelength confirms the inherent capability of the amino group to effectively induce red‐shift behavior.^[^ ^33^ ^]^ To further red‐shift the emission wavelength towards a lower energy range, we incorporated the lower electron density acceptor group naphtho[2,3‐c][1,2,5]selenadiazole into H_4_BTATC to form H_4_NSATC, employing it as the organic linker for the synthesis of HIAM‐4025, which emits with peak maxima at 788 nm (Figure 1c). Additionally, we compared the PL spectra of HIAM‐MOF and its organic ligand, as shown in Figure S2 (Supporting Information). We observed a slight blue shift in HIAM‐4023 compared to its organic ligand, which may be attributed to a more uniform dispersion of the organic ligand in the solution, leading to a weakening of π–π stacking and causing the blue shift. However, we observed that the PL fluorescence testing of HIAM‐4025′s organic ligand exhibited a weak fluorescence peak, indicating a less precise peak position and a relatively broad peak. In comparison to its organic ligand, HIAM‐4025 showed a subtle red shift. This phenomenon may be attributed to the constraints imposed on molecular vibrations and rotations within the framework after the formation of the MOF, resulting in a slight red shift in the spectral peak. The solid‐state photoluminescence quantum yields (PLQY) for HIAM‐4023, HIAM‐4024, and HIAM‐4025 were determined to be 2.2%, 1.1%, and 0.3%, respectively. Density functional theory (DFT) calculations were applied to analyze the electronic structures of these organic linkers, providing theoretical support to evaluate the influence of amino groups and low electron density acceptor groups on the emission behavior of the designed linker (Figure 1d). Upon the introduction of a single amino group to H_4_ABTTC, yielding H_4_BTATC, a gradual increase in LUMO energies from −2.57 eV to −2.34 eV was observed, while a more pronounced increase in HOMO energies was noted, rising from −5.53 eV to −5.17 eV. Consequently, the energy gaps between the HOMO and LUMO were significantly reduced, decreasing from 2.96 eV for H_4_ABTTC to 2.83 eV for H_4_BTATC. When replacing the acceptor group benzo[c][1,2,5] thiadiazole with naphtho [2,3‐c][1,2,5] selenadiazole, resulting in the formation of H_4_NSATC, a decrease of 0.4 eV in the LUMO energy and an increase of 0.16 eV in the HOMO energy were observed, leading to a decrease of 2.27 eV in the energy gap for H_4_NSATC, consistent with experimental findings.

**Figure 2:**
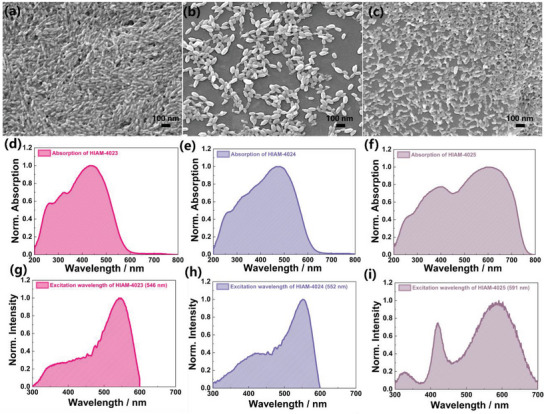
Scanning electron microscope (SEM), UV–vis absorption spectra, and the normalized solid‐state excitation spectrum of a,d,g) HIAM‐4023, b,e,h) HIAM‐4024, and c,f,i) HIAM‐4025.

The morphology of MOFs holds significance in evaluating their efficacy, particularly concerning perovskite films.^[^ ^34^, ^35^ ^]^ Achieving nanoscale morphology in MOFs has become pivotal for enhancing functionality and optimizing performance.^[^ ^36^, ^37^ ^]^ The scanning electron micrographs (SEM) of HIAM‐4023, HIAM‐4024, and HIAM‐4025 depicted in **Figure** **2**a–c highlight their nanosized (50‐70 nm) and rice‐like morphology. Additionally, the UV–visible absorption spectra reveal that HIAM‐4023 and HIAM‐4024 exhibit comparable absorption patterns, with the absorption band spanning 200–600 nm. In contrast, the spectrum of HIAM‐4025 exhibits a gradual bathochromic shift in its spectrum, with the absorption band extending from 200 to 800 nm, in line with their respective emission energies (Figure 2d–f). The peak values of the PL excitation spectrum were observed at wavelengths of 546, 552, and 591 nm, corresponding to the HIAM‐4023, HIAM‐4024, and HIAM‐4025 samples, respectively (Figure 2g–i). **Table** **1** provides a summary of the structures, absorption, excitation, emission properties, and quantum yield of synthesized HIAM‐MOFs.

**Table 1:** Summary of the organic linkers and absorption, excitation, and emission properties of the MOFs in this work.

organic linkers	MOFs	Emission wavelength [nm]	Absorption wavelength [nm]	Excitation wavelength [nm]
H_4_ABTTC	HIAM‐4023	676	200–600	546
H_4_BTATC	HIAM‐4024	688	200–600	552
H_4_NSATC	HIAM‐4025	788	200–800	591

In the investigation of potential energy transfer between the three HIAM‐MOFs and perovskite, it is considered the distinctions between FRET, NSET, and NVET.^[^ ^38^ ^]^ The choice of FRET over NSET and NVET is motivated by its unique advantages for the MOF and perovskite systems.^[^ ^17^, ^39^ ^]^ First, FRET is particularly well‐suited for describing energy transfer at closer proximity distances. NSET and NVET often rely on longer for efficient energy transfer.^[^ ^40^, ^41^ ^]^ Second, FRET efficiency is strongly spectral overlap, allowing for fine‐tuning of energy transfer efficiency by designing the spectral overlap of the fluorescent system.^[^ ^42^ ^]^ Last, FRET provided a more suitable energy transfer between HIAM‐MOFs and perovskite.^[^ ^43^ ^]^ Therefore, considering the advantages of FRET over NSET and NVET, we have chosen FRET as the primary mechanism to explore the potential occurrence of energy transfer between the three HIAM‐MOFs and perovskite.

**Figure 3:**
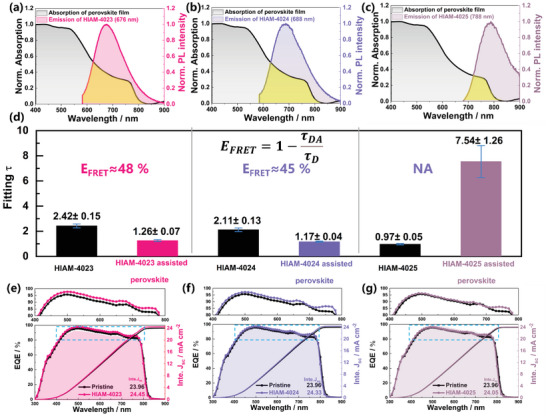
Spectral overlap between the PL emission spectrum of HIAM‐MOFs and the absorption spectra of perovskite for a) HIAM‐4023, b) HIAM‐4024, and c) HIAM‐4025. d) The bar chart depicts the average of the final fitted lifetimes from five repetitions of TRPL for each of the six materials. Error bars represent the standard deviation of five measurements for each film. The external quantum efficiencies (EQE) of the pristine, e) HIAM‐4023, f) HIAM‐4024, and g) HIAM‐4025‐assisted PSCs.

To establish the potential occurrence of FRET between the three HIAM‐MOFs and perovskite, a crucial requirement is the spectral overlap between the PL emission spectrum of HIAM‐MOFs and the absorption spectrum of perovskite.^[^ ^15^, ^44^ ^]^ This criterion ensures the efficient transfer of energy from the excited state of the HIAM‐MOFs to the perovskite, allowing for the exploration of energy transfer mechanisms and the evaluation of their influence on the optoelectronic properties and performance of hybrid systems. To support the occurrence of the FRET mechanism, we first performed an in‐depth examination of the spectral overlap between HIAM‐MOF and perovskite. Spectral overlap exists between the excitation spectra of the three HIAM‐MOFs and the absorption spectrum of the perovskite, albeit with varying overlap areas (**Figure** **3**a–c). Notably, substantial spectral overlap was observed between the PL excitation spectra of HIAM‐4023 and HIAM‐4024 and the perovskite absorption spectra. However, due to different acceptor units, HIAM‐4025 exhibited a pronounced shift towards the near‐infrared region, resulting in a substantially reduced overlap between the excitation spectrum of HIAM‐4025 and the absorption spectrum of the perovskite. The extent of spectral overlap is quantitatively assessed by the integration of overlap (*J(λ)*), as determined by the calculation described in Equation (1):^[^ ^45^ ^]^

(1) $$J\left(\lambda \right)=\frac{\int_{0}^{\infty }F_{D}\left(\lambda \right)\varepsilon_{A}\left(\lambda \right)\lambda^{4}d\left(\lambda \right)}{\int_{0}^{\infty }F_{D}\left(\lambda \right)d\left(\lambda \right)}$$

where *F*_D_(λ) represents the normalized fluorescence intensity of the HIAM‐MOF emission (such as $\int_{0}^{\infty }F_{D}\left(\lambda \right)d\left(\lambda \right)$= 1). ε_A_(λ) represents the molar absorption coefficient of the perovskite (M^−1^ cm^−1^). λ represents the wavelength of overlap (nm). We quantified the degree of overlap between the PL excitation spectra of HIAM‐4023, HIAM‐4024, and HIAM‐4025 and the perovskite absorption spectrum employing Equation 1, calculating the overlap integral of 6.63 × 10^15^, 6.50 × 10^15^, and 3.41 × 10^15^ M^−1^ cm^−1^ nm^4^ for HIAM‐4023, HIAM‐4024, and HIAM‐4025, respectively. This result emphasizes the impact of amino group incorporation and low electron density acceptor units on the PL properties of HIAM‐MOFs, leading to a significant red‐shift in the PL excitation peak towards the near‐infrared region and a gradual reduction of the spectral overlap with the perovskite absorption spectrum.

Additionally, the measurement of fluorescence lifetimes, which serves as a reliable indicator of energy transfer and can be utilized for determining FRET efficiencies, is another crucial component of evaluating the occurrence of FRET between HIAM‐MOFs and perovskites.^[^ ^46^, ^47^ ^]^ Therefore, we conducted a comprehensive investigation of the fluorescence lifetimes of HIAM‐MOFs and HIAM‐MOF‐assisted perovskites using a time‐resolved fluorescence spectrometer. This technique allowed us to investigate the complex dynamics of fluorescence emission in the presence of perovskite and to precisely determine the fluorescence lifetimes associated with energy transfer processes between HIAM‐MOFs and perovskites.^[^ ^48^, ^49^ ^]^ Comparatively, the fluorescence lifetimes of the HIAM‐4023 and HIAM‐4024‐assisted perovskite films decrease in comparison to HIAM‐MOFs alone, indicating effective energy transfer from HIAM‐4023 and HIAM‐4024 to the perovskite, thereby improving the photonic utilization efficiency of the perovskite. In contrast, the HIAM‐4025‐assisted perovskite films exhibited a significantly prolonged fluorescence lifetime, similar to the perovskite. This phenomenon can be attributed to the overlap between the photoluminescence emission peaks of HIAM‐4025 and the perovskite material. Consequently, our assessment suggests that the occurrence of FRET between HIAM‐4025 and the perovskite is unlikely. The FRET efficacy serves as the proportion of excited‐state energy transferred from the donor molecule to the acceptor molecule, quantifying the following Equation 2.^[^ ^50^ ^]^

(2) $$E_{\mathrm{FRET}}=\;1-\frac{\tau_{\mathrm{DA}}}{\tau_{D}}$$

where τ_D_ and τ_DA_ represent the fluorescence lifetime of the HIAM‐MOFs with and without the perovskite, respectively. Due to the uncertainties in TRPL experiments, we conducted five repetitions of TRPL experiments for each material, including HIAM‐4023, HIAM‐4024, and HIAM‐4025, along with their respective perovskite‐assisted thin films. The fluorescence lifetime decay curves, fitted lifetimes, and experimental instrument response function (IRF) can be found in Figure S3 (Supporting Information). Subsequently, we performed an analysis of the average and error bars for the TRPL fitting data of each film. The final fitting results are presented in Figure 3d. The average lifetimes of HIAM‐4023, HIAM‐4024 and HIAM‐4025 were measured to be 2.42, 2.11 and 0.97 ns, respectively. Upon the inclusion of the perovskite materials, these lifetimes notably decreased or increased to 1.26, 1.17 and 7.54 ns, respectively. *E*_FRET_ was calculated using Equation 2, yielding an energy transfer efficiency of 48% from HIAM‐4023 to the perovskite and 45% from HIAM‐4024 to the perovskite, indicating effective energy transfer from HIAM‐4023 and HIAM‐4024 to the perovskite. Additionally, no FRET occurs between HIAM‐4025 and the perovskite. Furthermore, our investigation into HIAM‐4023 and HIAM‐4023‐assisted perovskite films via femtosecond transient absorption spectroscopy (fs‐TAS) has yielded insightful results, as depicted in Figure S4 (Supporting Information). The pseudocolor representations (Figure S4a,b, Supporting Information) reveal a pronounced absorption feature near 620 nm for HIAM‐4023. Detailed temporal analysis, extracted from Figure S4d (Supporting Information) and displayed in Figure S4c (Supporting Information), indicates a time‐dependent quenching of the ≈620 nm fluorescence peak, converging back to baseline and signaling a photophysical decay pathway. Notably, the perovskite's absorption peak around 760 nm exhibits a discernible redshift, which may suggest an energy transfer from the HIAM‐MOF to the perovskite.^[^ ^51^, ^52^ ^]^ Selective probing at the ground state bleach peak of 620 nm allowed us to ascertain decay lifetimes (Figure S4d, Supporting Information), revealing a shortened lifetime within the HIAM‐4023‐facilitated perovskite system. This observation aligns with our TRPL findings and unequivocally supports the occurrence of an efficient FRET process between the HIAM‐4023 and the perovskite.

To further validate the occurrence of FRET, the distance parameter between HIAM‐MOF and the calcium titanium oxide becomes crucial. Typically, a distance range of 10–100 Å between the donor and acceptor is required to ensure that energy transfer can take place between them.^[^ ^53^, ^54^ ^]^ The donor‐acceptor distance (r) and Förster distance (R_0_) between HIAM‐MOF and perovskite were calculated in Equations (3) and (4):^[^ ^55^ ^]^

(3) $$R_{0}=0.211{\left(K^{2}n^{-4}Q_{D}J\left(\lambda \right)\right)}^{1/6}$$

(4) $$E=1/1+{\left(r/R_{0}\right)}^{6}$$

where Förster distance (*R*_0_, in Å) is defined as the separation distance (R) between a single pair involved in FRET that corresponds to an energy transfer efficiency of 50%. *Q*_D_ is HIAM‐MOFs quantum yield, *K* ^2^(orientation factor) is the orientation of the transition dipole moment of the FRET pair and is typically assumed to be 2/3, and *n* is the refractive index of the medium (perovskite *n* ≈ 2.5).^[^ ^56^, ^57^ ^]^ We calculated the distances between HIAM‐4023 and HIAM‐4024 with the perovskite to be 24.80 and 22.46 Å, respectively, satisfying the requirements for FRET to occur. **Table** **2** supplies an exhaustive summary of the specific parameters characterizing FRET between HIAM‐MOFs and perovskite.

**Table 2:** Summary of calculated FRET parameters between HIAM‐MOF and perovskite.

	*R*_0_ [Å]	*r* [Å]	*E* [%]
HIAM‐4023: perovskite	24.56	24.80	48
HIAM‐4024: perovskite	21.81	22.46	45
HIAM‐4025: perovskite	15.77	NA	NA

Upon the previous discussion, we have confirmed the existence of FRET between HIAM‐4023, HIAM‐4024, and perovskite, leading to increased light energy utilization in the perovskite films with improved short‐circuit current density (*J*_sc_), as evidenced by the enhanced external quantum efficiency (EQE) of the perovskite films. Specifically, HIAM‐4023 and HIAM‐4024 efficiently transfer their energy to the perovskite upon excitation, substantially increasing light absorption within the photoactive layer. This correlates with a notable EQE enhancement at approximately 600 nm. Incorporation of HIAM‐4023 resulted in EQE increasing from 23.96% to 24.45% (Figure 3e), while HIAM‐4024 led to EQE rising from 23.96% to 24.33% (Figure 3f), indicating relatively lower FRET conversion efficiency. Conversely, HIAM‐4025 did not exhibit Förster‐type transfer to the perovskite, resulting in comparable EQE values of 23.96% and 24.05% (Figure 3g). The enhanced EQE curve between 700 and 800 nm for three samples is attributed to the enhanced crystal quality of the perovskite film.^[^ ^30^, ^58^ ^]^

**Figure 4:**
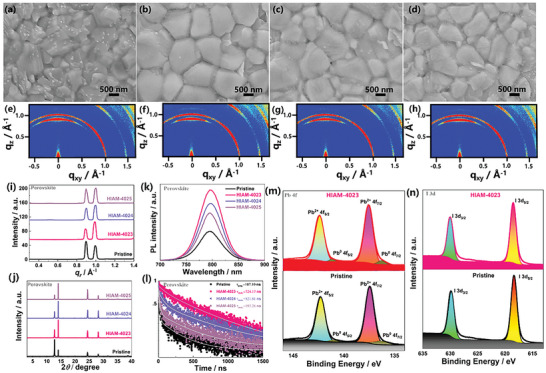
The plan‐view SEM and GIWAXS of the a,e) pristine, b,f) HIAM‐4023, c,g) HIAM‐4024, and d,h) HIAM‐4025‐assisted perovskite thin films. i) Radial integration corresponding GIWAXS patterns and j) XRD. k) Steady‐state PL and l) TRPL spectra of the pristine, HIAM‐MOF‐assisted perovskite films. XPS regional analysis of m) Pb 4f and n) I 3d for pristine and HIAM‐4023‐assisted PbI_2_ films.

Subsequently, we performed a thorough analysis of the effect of HIAM‐MOF on both PbI_2_ and perovskite films. Nanoscale HIAM‐MOF was introduced into the PbI_2_ solution to enable the fabrication of HIAM‐MOF‐assisted PbI_2_ thin film during the initial deposition step. The addition of HIAM‐MOFs caused a visible change in the precursor solution's appearance, transitioning from clear to turbid with distinct color variations (Figure S5, Supporting Information). The crystalline structure and orientation of PbI_2_ film remained unchanged upon the introduction of HIAM‐MOFs, as confirmed by grazing‐incidence wide‐angle X‐ray scattering (GIWAXS) and XRD (Figure S6, Supporting Information). UV–vis absorption spectra of both pristine and HIAM‐MOFs‐modified PbI_2_ thin films showed no alterations (Figure S8a, Supporting Information). To unveil additional insights into the optical and electronic properties of pristine and HIAM‐MOF‐assisted perovskite film, we employed plan‐view SEM to investigate the morphological impact of HIAM‐MOF on the perovskite film (**Figure** **4**a–d). The incorporation of HIAM‐MOF has significantly increased the perovskite grain size, which may be attributed to a slower nucleation rate, providing a more controlled and favorable growth environment for the perovskite crystals.^[^ ^59^, ^60^ ^]^ Additionally, in comparison to the pristine one (Figure 4e), the introduction of HIAM‐MOF leads to a significant reduction in intensity observed for the scattering ring at a low *q* value of 0.9 Å^−1^ (PbI_2_ phase), as clearly demonstrated in the GIWAXS maps (Figure 4f–h). Figure 4i illustrates the radial integration curves derived from the corresponding GIWAXS patterns to elucidate this finding in greater detail. The HIAM‐MOF‐assisted perovskite film demonstrates a distinct scattering ring at *q* = 1.0 Å^−1^ and a significantly suppressed scattering ring at *q* = 0.9 Å^−1^ in contrast to the pristine, corroborating the XRD data (Figure 4j). This concordance between the GIWAXS and XRD data further validates the effect of HIAM‐MOF on the structural properties of the perovskite film, highlighting its potential for enhanced phase purity and crystallinity.^[^ ^61^, ^62^, ^63^ ^]^ Additionally, to verify the integrity of HIAM‐MOF within the perovskite, an excess of HIAM‐MOF material was introduced into PbI_2_, and perovskite layers were prepared. Through XRD analysis (Figure S7, Supporting Information), we observed small peaks around 2*θ* ≈ 2.5° in all three HIAM‐MOF‐assisted perovskites, consistent with the XRD peaks of pure HIAM‐MOF in Figure 1b. This suggests that the structure of HIAM‐MOF is not disrupted during the solution processing of perovskite, confirming its integrity within the perovskite materials. The enhanced UV–vis absorption of perovskite films with HIAM‐MOFs was attributable to the efficient FRET process (Figure S8b, Supporting Information). Steady‐state photoluminescence (PL) (Figure 4k) results exhibited that the incorporation of HIAM‐4023 into the perovskite film increased PL intensity, pointing to effective nonradiative combination suppression, as confirmed by time‐resolved photoluminescence (TRPL) (Figure 4l). Fitting the experimental data of TRPL with the bi‐exponential function and the fitting parameters are summarized in Table S2 in Supporting Information. The average carrier lifespan of the HIAM‐4023‐assisted perovskite film (724.17 ns) was significantly longer than that of the pristine film (107.1 ns), confirming an effective FRET provided by the HIAM‐4023. Moreover, X‐ray photoelectron spectroscopy (XPS) was employed to investigate the elemental composition and chemical states of the pristine and HIAM‐MOFs‐assisted Pb_2_ film. The entire, S 2p, and Se 3d XPS spectra survey are given in Figure S9 (Supporting Information). The peak of Pb 4f and I 3d orbitals towards higher binding energies in the HIAM‐MOFs‐assisted PbI_2_ film (Figure 4m,n and Figure S10, Supporting Information), indicating interactions occurring between HIAM‐MOFs and the Pb^2+^ and I^−^ elements with strengthened bonding and charge redistribution.^[^ ^64^, ^65^ ^]^

**Figure 5:**
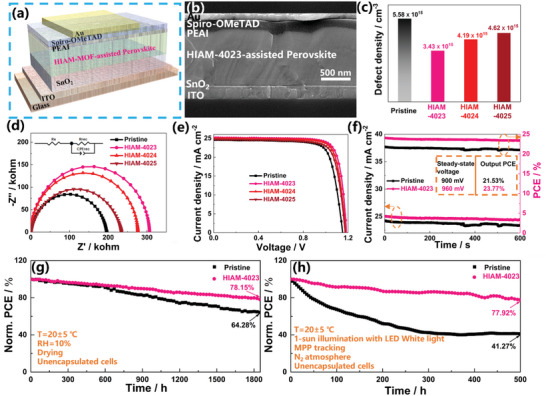
a) Schematic of PSCs and b) cross‐sectional SEM micrographs with a structure of ITO/SnO_2_/ HIAM‐4023‐assisted perovskite/PEAI/Spiro‐OMeTAD/Au. c) Trap density (*N*_t_), d) Nyquist plot of the EIS of the PSCs based on pristine, HIAM‐4023, HIAM‐4024, and HIAM‐4025. e) Current density–voltage (*J–V*) curves under an AM1.5G solar simulator for devices. f) The stabilized power outputs of the pristine and HIAM‐4023‐assisted devices were measured at voltages of 0.90 and 0.96 V in the ambient environment, respectively. g) Normalized PCEs of nonencapsulated devices exposed to dry conditions (10% relative humidity) in the dark. h) Stability tests of unencapsulated solar cell devices exposed to continuous illumination (1 sun) near the maximum power point with a white LED lamp at 20±5 °C in a nitrogen atmosphere.

Subsequently, we investigated PSCs with a standardized configuration of ITO/ SnO_2_/HIAM‐4023‐assisted perovskite/PEAI/Spiro‐OMeTAD/Au (**Figure** **5**a), as endorsed by the cross‐sectional SEM micrographs shown in Figure 5b. The pristine devices demonstrate comparable cross‐sectional structures, with noticeable differences in the perovskite grain size (Figure S11, Supporting Information). Trap densities (*N*_t_) of the devices were quantitatively evaluated using space‐charge limited current (SCLC) analyses, which were carefully fabricated on hole‐only ITO/PEDOT:PSS/perovskite/spiro‐OMeTAD/Au devices, as shown in Figure 5c and Figure S12 (Supporting Information). Notably, the *N*_t_ values were determined to be 5.58 × 10^16^  cm^−3^ for the pristine device, while significantly lower *N*_t_ values of 3.43 × 10^15^, 4.19 × 10^15^, and 4.62 × 10^15^ cm^−3^ were obtained for the HIAM‐4023, HIAM‐4024, and HIAM‐4025 assisted devices, respectively. The inclusion of HIAM‐4023 effectively suppresses the formation of planar trap states, reduces trap densities, and improves charge carrier transport properties within the perovskite material. Furthermore, the HIAM‐4023‐assisted device demonstrates a significantly elevated built‐in potential of 1.06 V compared with the pristine (0.91 V) (Figure S13, Supporting Information), providing compelling evidence that the incorporation of HIAM‐4023 effectively enhances charge transport and mitigates recombination losses at the interface.^[^ ^66^ ^]^ The HIAM‐4023‐assisted cells have a greater charge recombination resistance (*R*_rec_) than pristine, HIAM‐4024‐assisted, and HIAM‐4025‐assisted cells, which can be attributed to the low levels of defects in the perovskite layers (Figure 5d).

The current density–voltage (*J–V*) profiles for varying concentrations of HIAM‐4023 are shown in Figure S14 (Supporting Information), with Table S3 (Supporting Information) providing a comprehensive summary of the photovoltaic parameters. The HIAM‐4023‐assisted cell achieves a notable power conversion efficiency (PCE) of 24.22% (Figure 5e), with a *J*_sc_ of 25.16 mA cm^−2^, an open‐circuit voltage (*V*_oc_) of 1.184 V, and a fill factor (FF) of 81.31%, which is higher than that of the pristine and other assisted devices (Table S4, Supporting Information). The enhancements in FF and *V*_oc_ are attributable to the incorporation of HIAM‐4023, which improves the crystal quality of the perovskite film by promoting the formation of larger particle sizes and suppressing the formation of defect states. The increase in *J*sc is due to FRET between HIAM‐4023 and perovskite material, consistent with a previous discussion regarding the enhancement of EQE. The steady‐state output powers, determined at the maximum power point voltages, yield values of 21.53% and 23.77% for the pristine and HIAM‐4023‐assisted devices, respectively (Figure 5f). To evaluate the potential impact of HIAM‐4023‐assisted on the long‐term durability of PSCs, a series of stability studies were conducted on unencapsulated devices exposed to varying environmental conditions. The HIAM‐4023‐assisted device revealed remarkable long‐term stability, retaining 78.15% of its original PCE over 1800 h of exposure to dry environments (10% relative humidity). In contrast, the PCE of the pristine device degraded significantly by 35.72% under the same circumstances (Figure 5g). Meanwhile, when exposed to constant irradiation of 100 mW cm^−2^ LED white light with an LED solar simulator (model SLS‐LED‐80) for 500 h, the HIAM‐4023‐assisted cell retained 77.92% of its initial PCE, while the original device retained only 41.27% of its initial PCE (Figure 5h). These results demonstrate the significant stability advantage of the HIAM‐4023‐assisted device, confirming its superiority in a variety of environmental conditions.

## 3 Conclusion

In summary, we have successfully synthesized nanoscale HIAM‐type Zr‐MOF materials (HIAM‐4023, HIAM‐4024, and HIAM‐4025) with adjustable emission wavelengths by designing distinct organic linkers. The integration of these linkers with Zr_6_ clusters yielded well‐defined MOF structures with tunable photoluminescence properties. Remarkably, the HIAM‐4023 and HIAM‐4024‐assisted perovskite cells exhibit enhanced EQE through FRET processes, while HIAM‐4025 demonstrates comparable EQE to pristine devices. Moreover, the HIAM‐MOF‐assisted perovskite thin films exhibit enhanced quality and full PbI_2_‐to‐perovskite conversion. Notably, the HIAM‐4023‐assisted photovoltaic device achieves a high PCE of 24.22% compared with pristine cells (22.06%), demonstrating improved stability under ambient conditions and continuous LED light exposure. The ability to tailor MOF structures with precise control over their composition and topology offers exciting opportunities for enhancing energy transfer processes in perovskite‐based devices.

## Conflict of Interest

The authors declare no conflict of interest.

## Supporting information

Supporting Information

## Data Availability

Research data are not shared.
