# Supplementary material for: Facile Tailoring of Metal‐Organic Frameworks for Förster Resonance Energy Transfer‐Driven Enhancement in Perovskite Photovoltaics
Source: Adv Sci (Weinh). 2024 Mar 6;11(18):2307476. doi: 10.1002/advs.202307476 (PMC11095144; doi:10.1002/advs.202307476)
Supplement: Supplementary file 1 — Supporting Information [file ADVS-11-2307476-s001.pdf]

ADVANCED SCIENCE
Open Access

Supporting Information

for *Adv. Sci.*, DOI 10.1002/advs.202307476

Facile Tailoring of Metal-Organic Frameworks for Förster Resonance Energy Transfer-Driven Enhancement in Perovskite Photovoltaics

Xiao Liang, Hai-lun Xia, Jin Xiang, Fei Wang, Jing Ma, Xianfang Zhou, Hao Wang, Xiao-Yuan Liu*, Quanyao Zhu*, Haoran Lin, Jun Pan, Mingjian Yuan, Gang Li* and Hanlin Hu**

Facile tailoring of Metal-Organic Frameworks for Förster Resonance Energy Transfer-Driven Enhancement in Perovskite Photovoltaics

Supporting Information

DOI: 10.1002/
Article type: Communication

Xiao Liang[‡1,2], Hai-lun Xia[‡1], Jin Xiang[‡1], Fei Wang[1,2], Jing Ma[3*], Xianfang Zhou[1,2], Hao Wang[1], Xiao-Yuan Liu[1*], Quanyao Zhu[2*], Haoran Lin[1], Jun Pan[5], Mingjian Yuan[6], Gang Li[4*], Hanlin Hu[1*]

[1]Hoffmann Institute of Advanced Materials, Shenzhen Polytechnic, 7098 Liuxian Boulevard, Shenzhen 518055, China.

[2]State Key Laboratory of Advanced Technology for Materials Synthesis and Processing, School of Materials Science and Engineering, Wuhan University of Technology, Wuhan, China.

[3]Medical Intelligence and Innovation Academy, Southern University of Science and Technology Hospital, 518055, China.

[4]Department of Electronic and Information Engineering, Research Institute for Smart Energy (RISE), The Hong Kong Polytechnic University, Hung Hom, Kowloon, Hong Kong, China.

[5]College of Materials Science and Engineering, Zhejiang University of Technology, Hangzhou, 310014 China

[6]Renewable Energy Conversion and Storage Center (RECAST) College of Chemistry, Nankai University, Tianjin 300071, China.

‡ X.L., H. X. and J. X contributed equally to this work.

Experimental Section

Materials: ITO glass substrates with a sheet resistance of ca. 14 Ω sq^{-1} were purchased from OPVTECH Inc. HIAM-4023, HIAM-4024, and HIAM-4025 were synthesized. Formamidinium Iodide (FAI) and methylammonium bromide (MABr) were supplied from Dyesol. Methylammonium chloride (MACl), bis (trifluoromethane) sulfonimide lithium salt (Li-TFSI, 99%), 4-tert-butylpyridine (tBP, 96%), Spiro-OMeTAD (purity. 99.5%), CsI (99.99%) were supplied from Xi'an Polymer Light Technology Corp. Tin (II) chloride dehydrate ($SnCl_2 \cdot 2H_2O$), thiourea, DMF, DMSO, CB, acetonitrile were purchased from Sigma-Aldrich. PbI_2 (99.8%) was purchased from TCI. All chemicals were used as received without further treatment.

Synthesis of HIAM-4023: 20.0 mg $ZrOCl_2 \cdot 8H_2O$, 10 mg H_4ABTTC linkers, 0.30 mL formic acid, and 3 mL DMF were added in a 5 mL vial. The mixture was sonicated for several minutes and then put into the 90°C preheated oven for 30 minutes. After cooling down to room temperature, the formed nano-MOFs were obtained by centrifugation at 11000 rpm for 10 minutes, which was washed using DMF and methanol for three times.

Synthesis of HIAM-4024: 20.0 mg $ZrOCl_2 \cdot 8H_2O$, 10 mg H_4BTATC linkers, 0.35 mL formic acid, and 3 mL DMF were added in a 5 mL vial. The mixture was sonicated for several minutes and then put into the 90°C preheated oven for 30 minutes. After cooling down to room temperature, the formed nano-MOFs were obtained by centrifugation at 11000 rpm for 10 minutes, which was washed using DMF and methanol for three times.

Synthesis of HIAM-4025: 20.0 mg $ZrOCl_2 \cdot 8H_2O$, 10 mg H_4NSATC linkers, 0.50 mL formic acid, and 3 mL DMF were added in a 5 mL vial. The mixture was sonicated for several minutes and then put into the 90°C preheated oven for 30 minutes. After cooling down to room temperature, the formed nano-MOFs were obtained by centrifugation at 11000 rpm for 10 minutes, which was washed using DMF and methanol for three times.

Devices Fabrications: The PSCs were fabricated on cleaned ITO substrates. ITO substrates were sequentially rinsed by sonication in detergent, deionized (DI) water, acetone, and ethanol, and finally dried in the air by nitrogen flow. Before the deposition of ETL, ITO substrates were exposed to UV–ozone for 30 min. A thin layer of SnO_2 nanoparticle film was spin-coated on the ITO substrate at 4,000 r.p.m. for 30 s to form a 50-nm-thick ETL and annealed in ambient air at 150 °C for 30 min.

Then, the perovskite layer was deposited on the SnO_2 layer by a two-step spin-coating method. The PbI_2-HIAM-MOF precursor solution was prepared by dissolving 1.4 M PbI_2 into the mixed solvent of DMF and DMSO (4.5:0.5, v/v) in a nitrogen-filled glove box. The dissolved solution was filtered with the filter (0.22μm, Oriental Chemicals). The filtered PbI_2 solution added HIAM-4023, HIAM-4024, and HIAM-4025 powder and sealed sonicated for 30 min. The PbI_2 precursor solution was first spin-coated on the glass at 1500 rpm for 30 s. The substrate with the newly deposited PbI_2 layer was annealed at 70 °C for 1 min. After the PbI_2 film cooled down to room temperature, 40 μL of the organic mixture solution of FAI: MACl: MABr (60: 6: 6 mg in 1 mL IPA) was spin-coated onto the PbI_2 during spinning at 1800 rpm for 30 s. When the resulting film turned from orange to dark brown in drying, they were thermally annealed at 130 °C for 30 min under ambient conditions. Filtered spiro-OMeTAD solution (72.3 mg dissolved in 1 mL chlorobenzene) with 30 μL of tBP and 35 μL of Li-TFSI (260 mg mL^{-1}in acetonitrile) was spin-coated on the top of the perovskite layer at 4000 rpm for 30 s in a glove box after the substrates cooling down to room temperature.

Finally, 90–100 nm of gold was deposited by thermal evaporation on top of the HTL layer to complete the device, using a shadow mask to pattern the electrodes. The active area of the cells was 0.09 cm^2, which was defined by the overlapped area of the Au electrode and the ITO stripe.

GIWAXS measurement: GIWAXS measurements were performed at the Synchrotron & Printable Electronic Lab, Hoffmann Institute of Advanced Materials, Shenzhen Polytechnic

with SAXSFocus 3.0 equipped with a Cu X-ray Source (8.05 keV, 1.54 Å) and a EIGER 2R 500K detector. The incidence angle is 0.5°.

Characterization: The *J–V* characteristics of the devices were measured using a B1500 A semiconductor parameter analyzer under the calibrated ABET Technologies SUN 2000 solar simulator equipped with an AM 1.5 filter at 100 mW cm^{-2}.

The corresponding IPCE spectrum was measured in air by a QE-R3011 system from Enli Technology Co. Ltd. (Enli).

The morphologies of PSCs were investigated by a high-resolution field emission SEM (JEOL JSM-6335F).

PL spectrum and TRPL signals of perovskite film were recorded by using Edinburgh FLSP920 spectrophotometer equipped with the excitation source of 465 nm picosecond pulsed diode laser.

Photoluminescence quantum yield (PLQY) was measured on a C9920-03 absolute quantum yield measurement system (Hamamatsu Photonics) with a 150 W xenon monochromatic light source and 3.3 inch integrating sphere.

Femtosecond transient absorption spectroscopy was conducted on the Shiyanjia Lab (www.shiyanjia.com) using the Ultrafast Helios pump-probe spectrometer provided by the company Ultrafast Systems. The laser source employed was the Coherent Astrella with a pulse energy exceeding 7mJ, a central wavelength of 800nm, pulse width less than 100fs, and a repetition rate of 1kHz.

Table S1. The recent summary report on the energy transfer occurring between MOFs and perovskites.

MOF	MOF-PL (nm)	perovskite	Energy transfer type	Year	Ref.
Bio-MOF-1	400-600	$MAPbBr_3$ quantum dots	FRET	2019	[1]
AMOF-1	420-580	$CsPbX_3$ quantum dots	FRET	2019	[2]
In-BTC	400-700	FAMACs	FRET	2020	[3]
Eu-MOF	570-720	FAMACs	FRET	2021	[4]
Eu-TCPP	625-800	$MAPbI_3$	FRET	2023	[5]
Y346	400-700	$MAPbX_3$ quantum dots	FRET	2023	[6]
Tb-cpon	480-630	FAMACs	FRET	2023	[7]

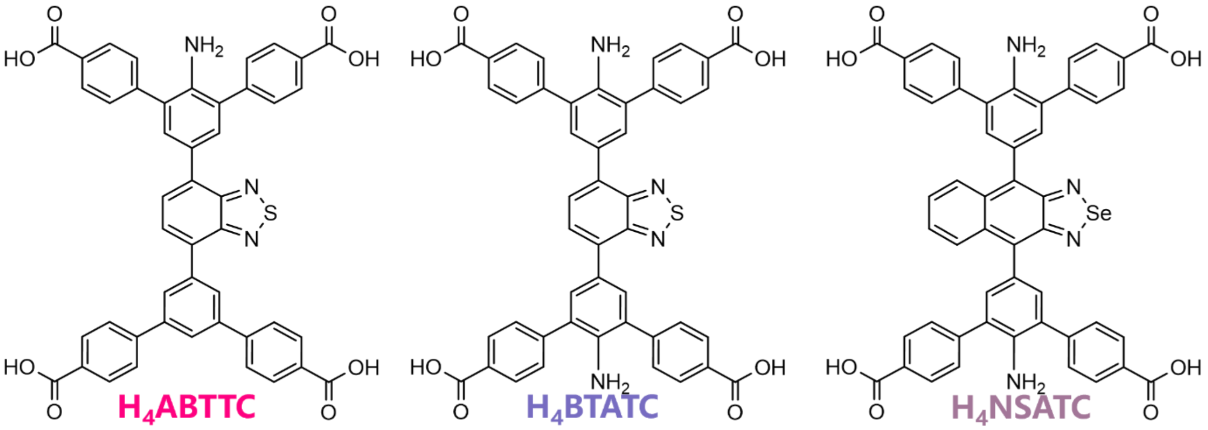

Figure S1. Molecular structures of the H_4ABTTC, H_4BTATC, and H_4NSATC.

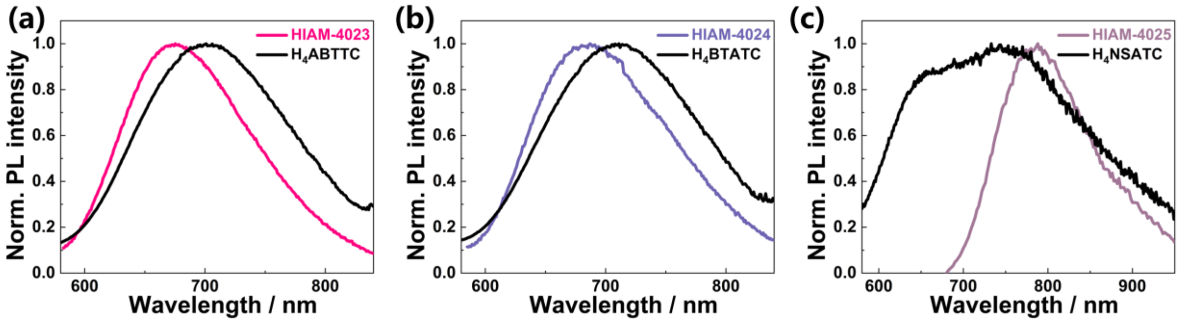

Figure S2. PL Spectrum of HIAM-MOF with its organic ligand.

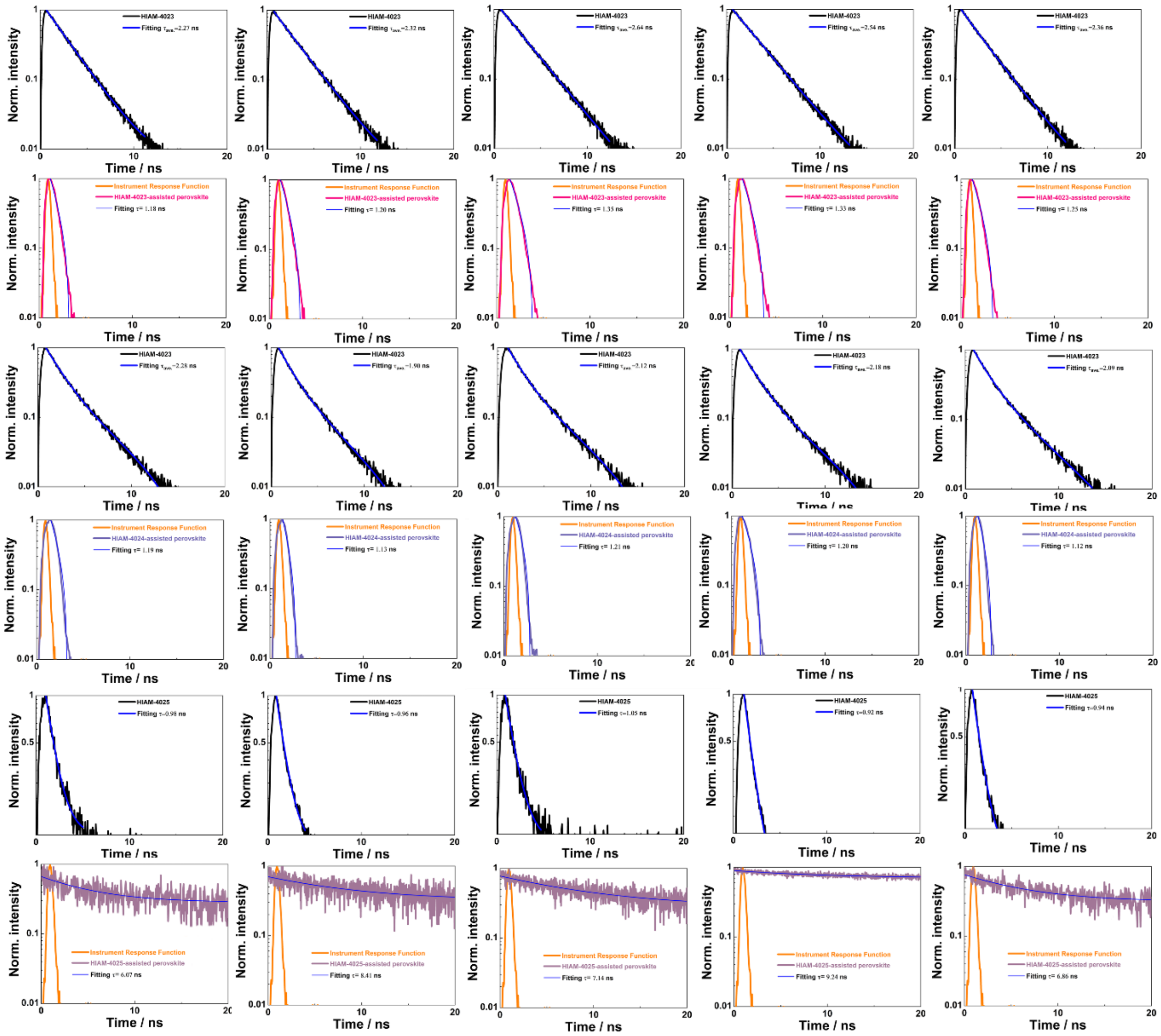

Figure S3. The fluorescence lifetime decay curves and fitting profiles of coexpressed HIAM-MOF with and without perovskite, in conjunction with the experimental instrument response function (IRF) for HIAM-4023, HIAM-4024, and HIAM-4025. (Repeated TRPL experiments were conducted five times for each of the six materials.)

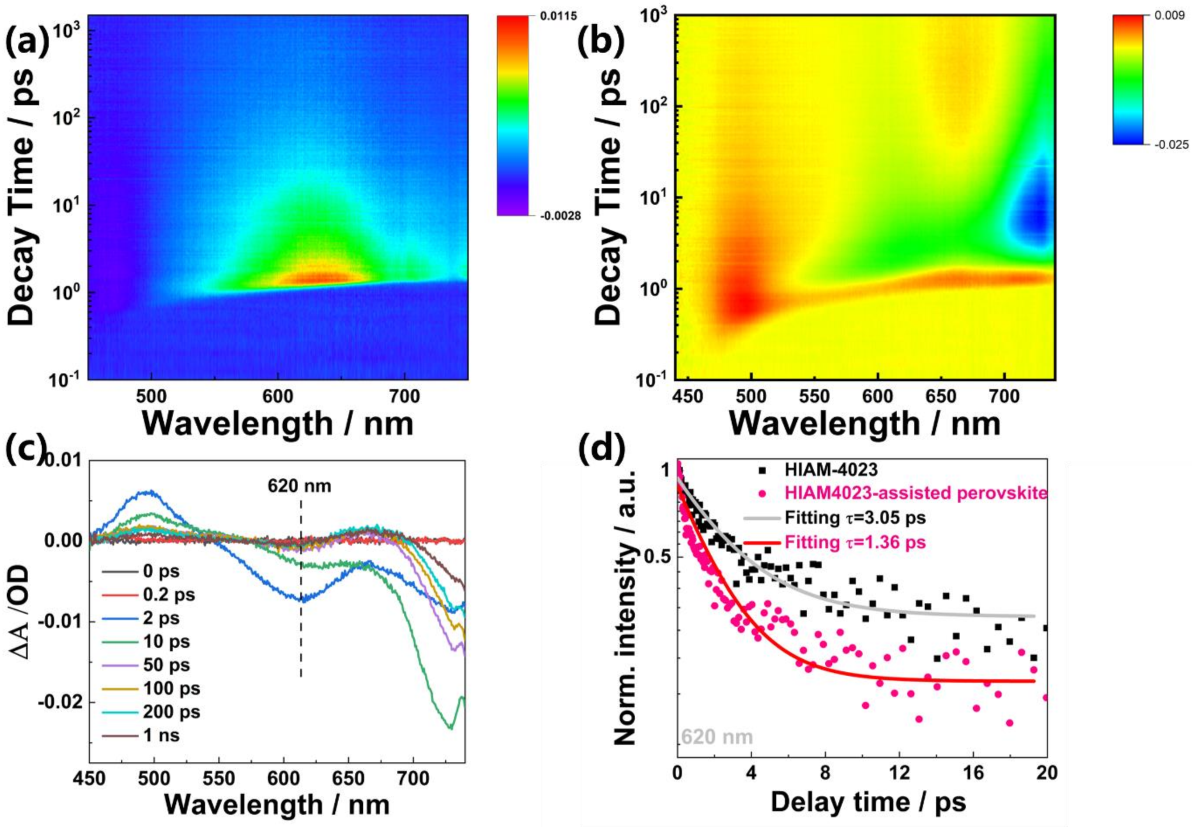

Figure S4. Pseudocolor plots showing the change in optical density (ΔOD) as a function of wavelength and decay time after excitation with a 400 nm laser pulse for (a) HIAM-4023 and (b) HIAM-4023-assisted perovskite, and (c) Femtosecond transient absorption spectroscopy (fs-TAS). (d) Normalized recovery kinetics of HIAM-4023 and HIAM-4023-assisted perovskite probed at 620 nm.

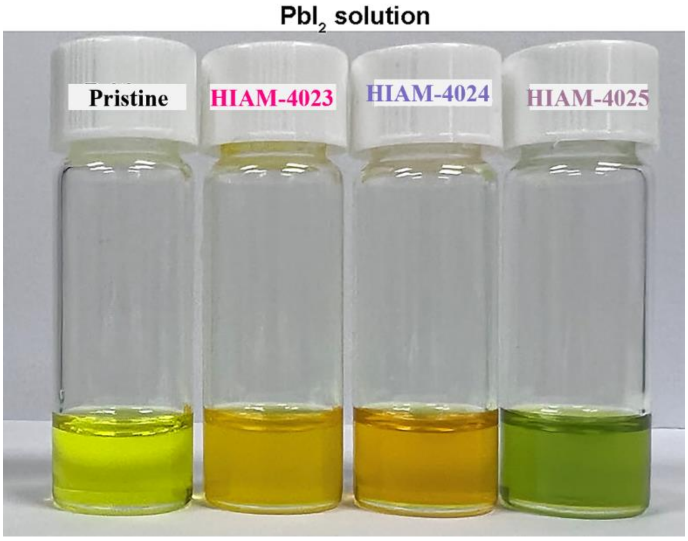

Figure S5. Photographs of the PbI_2 precursor solutions with pristine and HIAM-MOF-assisted.

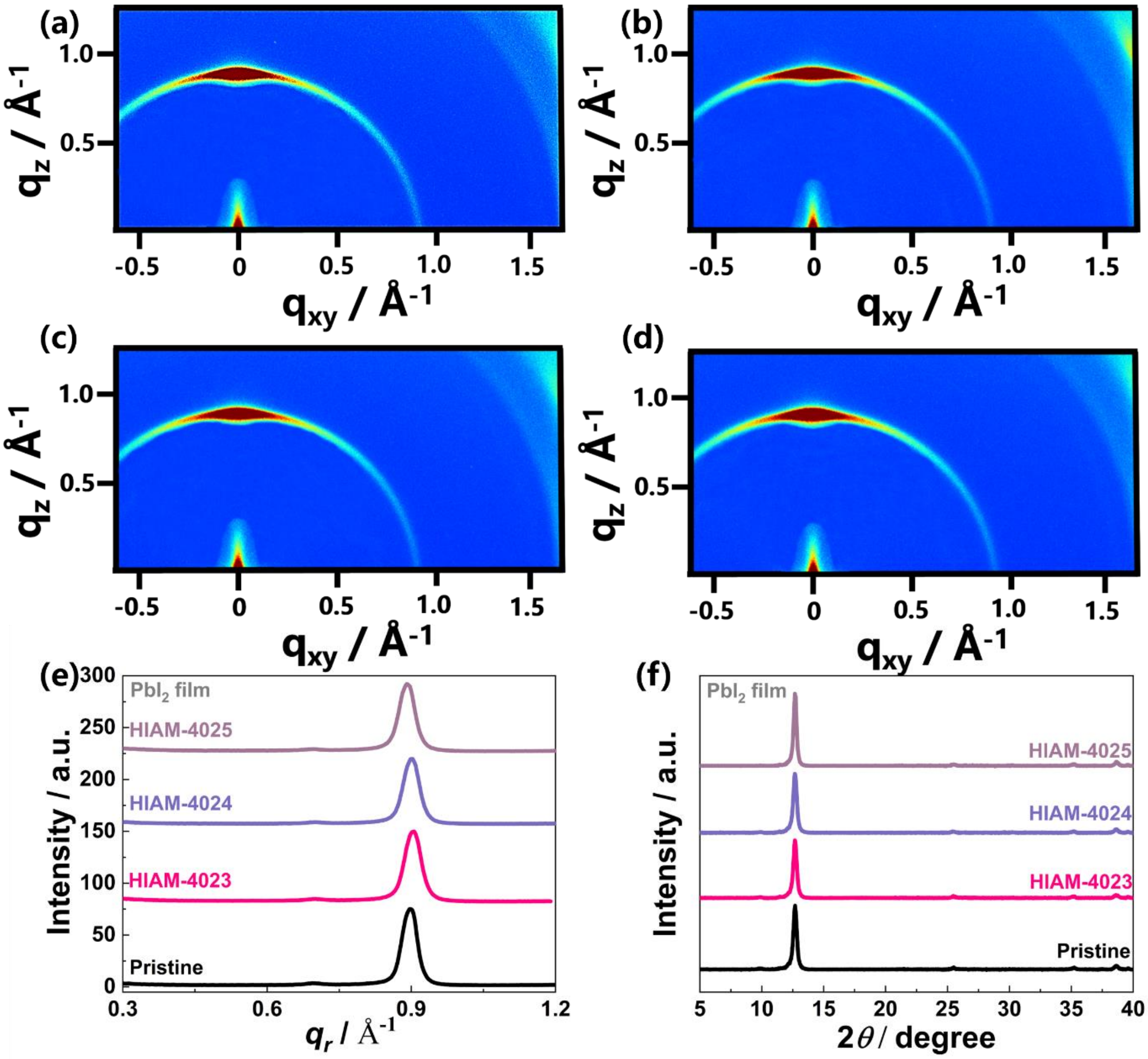

Figure S6. (a-d) Grazing-incidence wide-angle X-ray scattering (GIWAXS) patterns of the pristine and HIAM-MOFs assisted PbI_2 thin films. (e,f) XRD and Radial integration of PbI_2 from corresponding GIWAXS patterns.

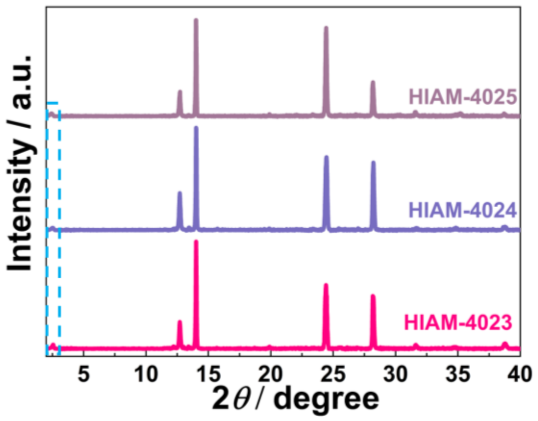

Figure S7. XRD of excess HIAM-MOF-assisted perovskite films.

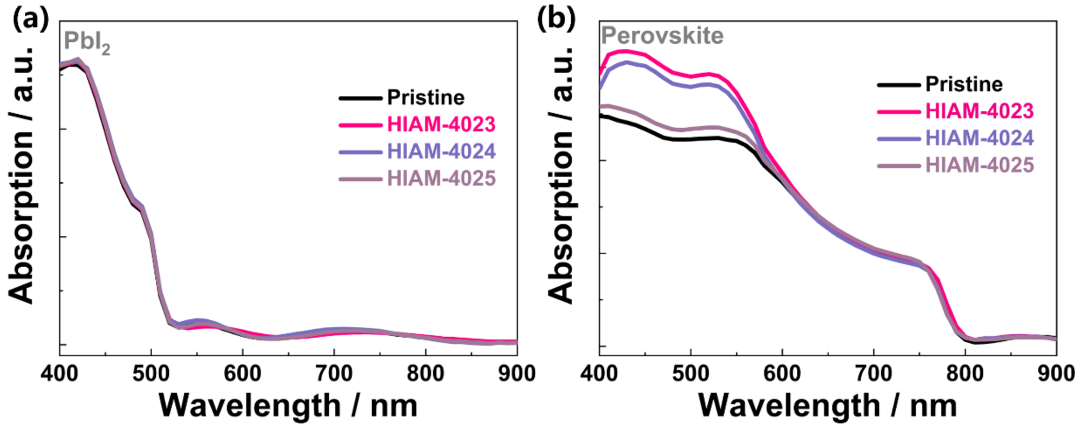

Figure S8. UV-vis of the pristine, HIAM-MOFs assisted (a) PbI_2 and (b) perovskite films.

Table S2. Fitting parameters of the bi-exponential decay function in TRPL spectra for the pristine and HIAM-MOFs assisted perovskite films

Sample	A_1	τ_1 / ns	A_2	τ_2 / ns	Average decay time τ / ns
pristine	0.45	14.94	0.18	337.50	107.1
HIAM-4023	0.13	119.08	0.63	849.04	724.17
HIAM-4024	0.23	72.24	0.37	801.27	521.81
HIAM-4025	0.43	19.26	0.24	505.03	193.26

Average decay time was calculated according to the equation: $\tau = (A_1\tau_1 + A_2\tau_2)/(A_1 + A_2)$.

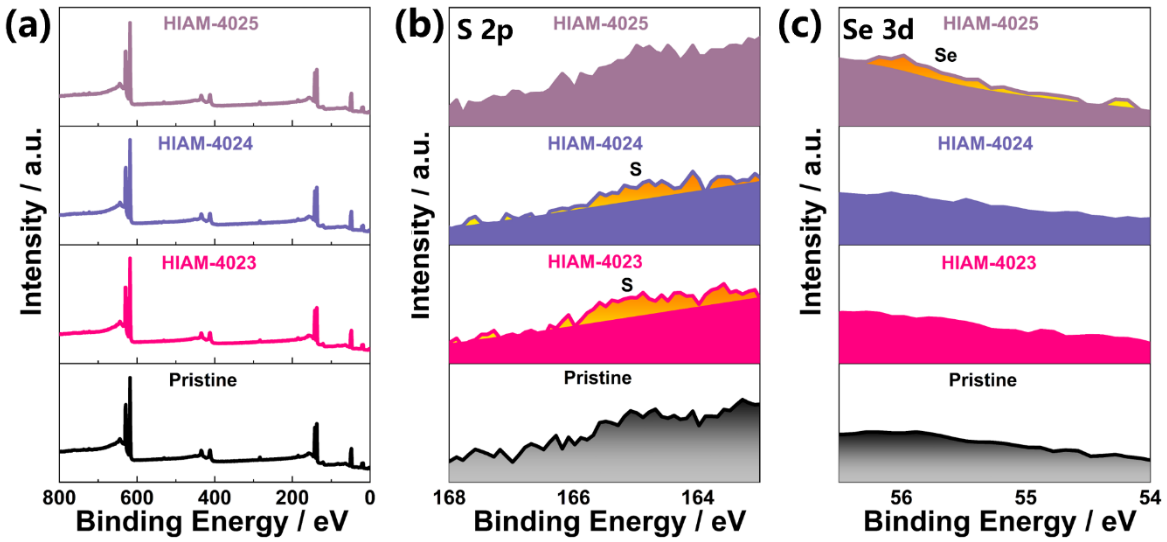

Figure S9. XPS measurements of (a) entire binding energy survey, (b) S 2p, and (c)Se 3d for pristine, HIAM-MOFs assisted PbI_2 film.

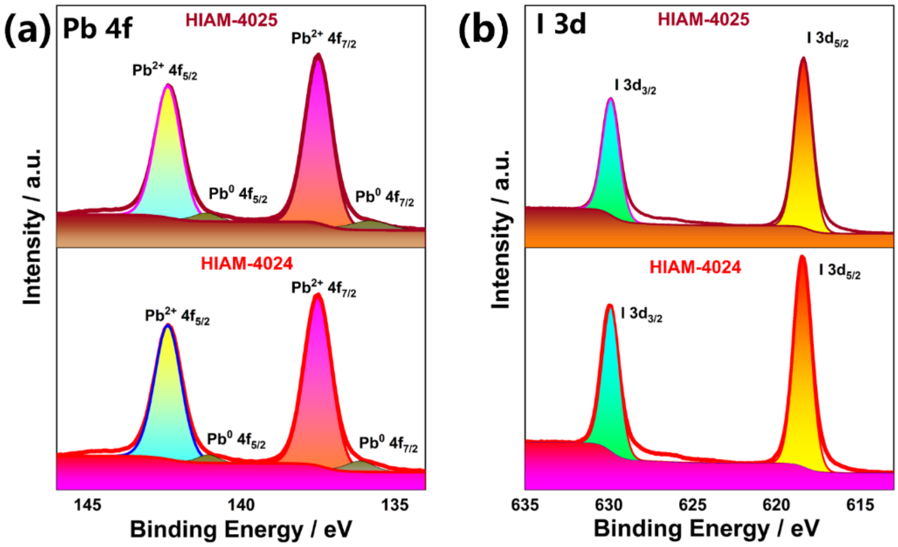

Figure S10. XPS regional analysis of (m) Pb 4f and (n) I 3d for pristine and HIAM-4024 and HIAM-4025-assisted PbI_2 films.

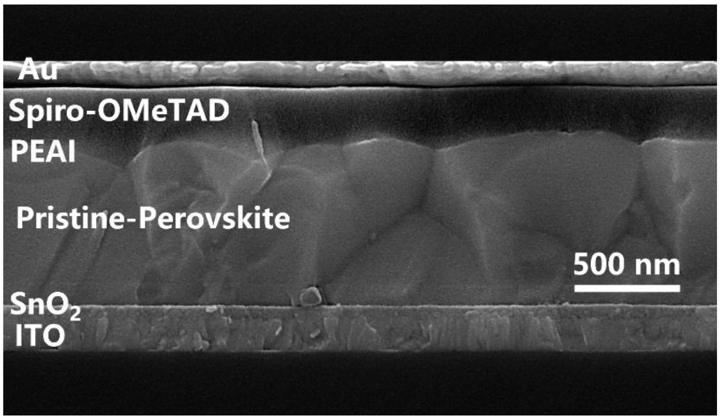

Figure S11. Cross-sectional SEM micrographs for pristine with a structure of ITO/SnO_2/perovskite/PEAI/Spiro-OMeTAD/Au.

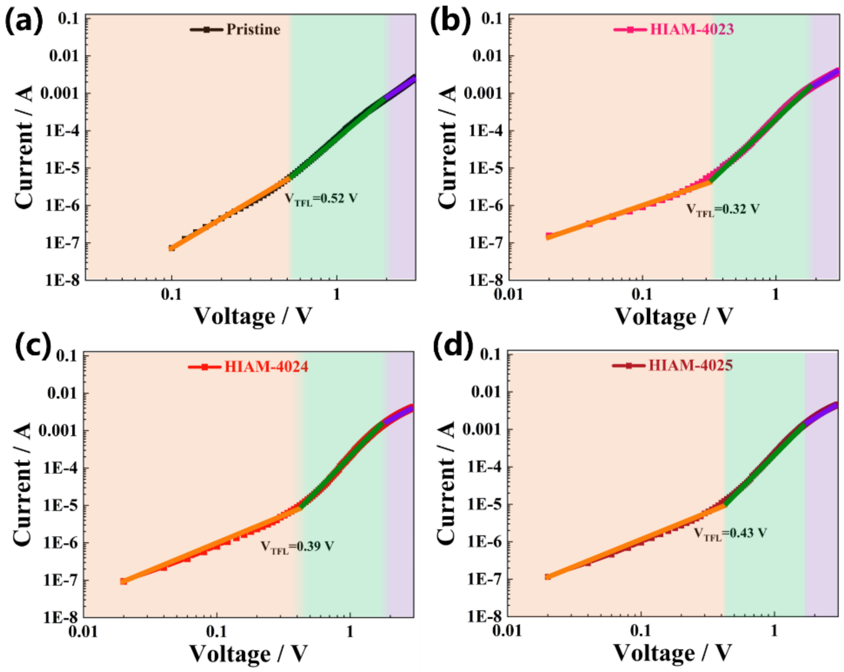

Figure S12.Space-Charge Limited Current indicates the trap-filling limited voltage (V_{TFL}) of the hole-only devices for (a) pristine, (b) HIAM-4023 assisted, (b) HIAM-4024 assisted, and (b) HIAM-4025 assisted.

The N_t of perovskite films was calculated using the SCLC method according to the following equation (5).

$$N_t = \frac{2\varepsilon\varepsilon_0 V_{TFL}}{eL^2} \tag{5}$$

Where e denotes elementary charge, L represents the thickness of the perovskite film, ε means the relative dielectric constant of perovskite, and ε_0 indicates the vacuum permittivity. V_{TFL} is the onset voltage of the trap-filled limit region.

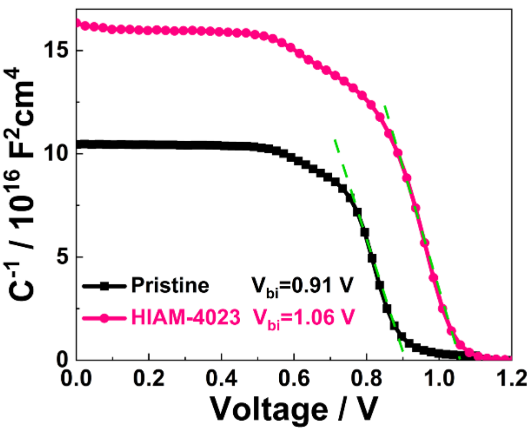

Figure S13. Mott–Schottky plots of the devices reveal the interfacial charge density for the pristine and HIAM-4023-assisted devices.

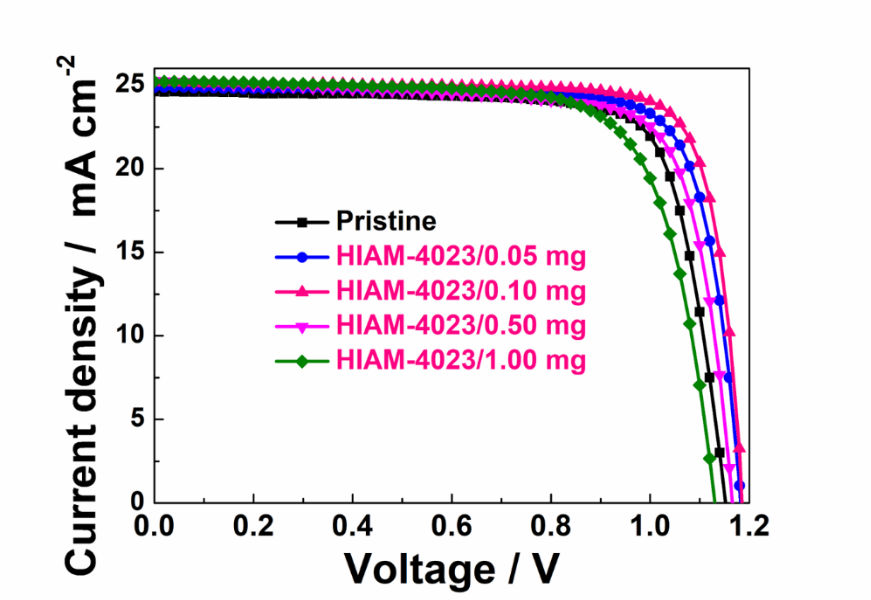

Figure S14. Current density–voltage (*J-V*) curves of the pristine and different concentrations of HIAM-4023-assisted devices.

Table S3. Photovoltaic parameters of PSCs with control and different concentrations of HIAM-4023 under AM 1.5G illumination at 100 mW cm^{-2}.

	Jsc (mA cm^{-2})	V_{oc} (V)	FF (%)	PCE (%)
Pristine	24.60	1.152	77.84	22.06
HIAM-4023/0.05 mg	24.87	1.178	79.36	23.25
HIAM-4023/0.10 mg	25.16	1.184	81.31	24.22
HIAM-4023/0.50 mg	25.17	1.165	76.57	22.45
HIAM-4023/1.00 mg	25.21	1.131	73.31	20.90

Table S4. Photovoltaic parameters of PSCs with pristine and HIAM-MOFs-assisted under AM 1.5G illumination at 100 mW cm^{-2}.

	Jsc (mA cm^{-2})	V_{oc} (V)	FF (%)	PCE (%)
Pristine	24.60	1.152	77.84	22.06
HIAM-4023-assisted cells	25.16	1.184	81.31	24.22
HIAM-4024-assisted cells	25.02	1.171	80.57	23.61
HIAM-4025-assisted cells	24.73	1.169	79.05	22.85

Reference

[1] Q. Zhang, H. Wu, W. Lin, J. Wang, Y. Chi, *J. Solid State Chem.* **2019**, *272*, 221.

[2] S. Bhattacharyya, D. Rambabu, T. K. Maji, *J. Mater. Chem. A* **2019**, *7*, 21106.

[3] X. Zhou, L. Qiu, R. Fan, J. Zhang, S. Hao, Y. Yang, *Nano-Micro Lett.* **2020**, *12*, 80.

[4] J. Dou, C. Zhu, H. Wang, Y. Han, S. Ma, X. Niu, N. Li, C. Shi, Z. Qiu, H. Zhou, Y. Bai, Q. Chen, *Adv. Mater.* **2021**, *33*, DOI 10.1002/adma.202102947.

[5] D. Wu, H. Zhou, X. Lai, X. Liu, K. Sang, Y. Chen, M. Chen, J. Wei, S. Wu, Q. Pang, L. Zhou, P. Chen, *Small* **2023**, DOI 10.1002/smll.202308783.

[6] Z. Yin, Q. Sun, J. Leng, L. Liu, B. Wu, S. Jin, *J. Phys. Chem. C* **2023**, *127*, 10655.

[7] W. Wang, J. Zhang, K. Lin, J. Wang, X. Zhang, B. Hu, Y. Dong, D. Xia, Y. Yang, *Adv. Mater.* **2023**, *35*, DOI 10.1002/adma.202306140.
